# A ‘fishy’ ECG in a patient with chest pain

**DOI:** 10.1007/s12471-021-01631-1

**Published:** 2021-09-15

**Authors:** V. Devesa Neto, J. M. Santos, J. G. Pereira, L. Ferreira Santos, B. Marmelo

**Affiliations:** Cardiology Department, Tondela-Viseu Hospital Centre, Viseu, Portugal

A 71-year-old man with a past medical history of diabetes mellitus and obesity presented to his primary care physician complaining of sudden-onset anterior chest pain, which radiated to the upper left arm and was associated with nausea. Hospital transportation was complicated by three short episodes of cardiac arrest in pulseless electrical activity/asystole, which reverted after appropriate advanced life support measures, and extreme bradycardia, requiring external transcutaneous pacemaker therapy. Sedation and ventilation were required during transportation. At hospital admission, the patient had a blood pressure of 90/85 mm Hg and a heart rate of 119 bpm, with signs of poor peripheral perfusion. The electrocardiogram (ECG) obtained at admission is shown in Fig. 1.

**Fig. 1:**
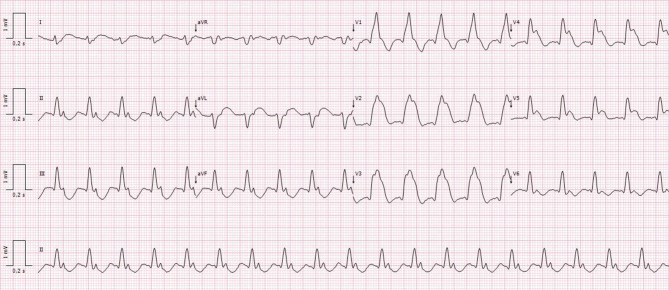
Electrocardiogram at hospital admission

How is this electrocardiography pattern called, and what lesion is it associated with?

## Answer

You will find the answer elsewhere in this issue.

